# Lignification of Sheepgrass Internodes at Different Developmental Stages and Associated Alteration of Cell Wall Saccharification Efficiency

**DOI:** 10.3389/fpls.2017.00414

**Published:** 2017-03-27

**Authors:** Jianli Wang, Lichao Ma, Zhongbao Shen, Dequan Sun, Peng Zhong, Zetao Bai, Hailing Zhang, Yingping Cao, Yan Bao, Chunxiang Fu

**Affiliations:** ^1^Grass and Science Institute of Heilongjiang Academy of Agricultural SciencesHarbin, China; ^2^Key Laboratory of Biofuels, Shandong Provincial Key Laboratory of Energy Genetics, Qingdao Institute of Bioenergy and Bioprocess Technology, Chinese Academy of SciencesQingdao, China; ^3^Qingdao Engineering Research Center of Biomass Resources and Environment, Qingdao Institute of Bioenergy and Bioprocess Technology, Chinese Academy of SciencesQingdao, China; ^4^Rural Energy Research Institute of Heilongjiang Academy of Agricultural SciencesHarbin, China

**Keywords:** cell wall, forage, lignification, saccharification, sheepgrass

## Abstract

Sheepgrass (*Leymus chinensis*) is a high-quality cool-season forage crop used as pasture and hay for livestock feeds. The presence of lignin in cell walls, however, impairs forage digestibility of such lignocellulosic feedstock. Here, the structural characterization and cell wall composition of sheepgrass internodes were studied, and a progressive increase in cell wall lignification was observed with internode maturation. Lignin composition analysis further revealed a gradual accumulation of guaiacyl and syringyl lignin units during internode development. Consistently, the transcript abundance of lignin-related genes was upregulated in mature internodes, suggesting their potential roles in lignin biosynthesis. Furthermore, the effects of cell wall composition and lignification extent on biomass saccharification efficiency were examined in sheepgrass. The results showed that lignin content, guaiacyl and syringyl lignin unit levels inversely correlated with cell wall digestibility, indicating that lignin is a crucial obstacle for utilizing sheepgrass feedstock. The baseline information obtained in this work will facilitate establishment, grazing management, harvesting and feedstock utilization of sheepgrass in future.

## Introduction

Sheepgrass (*Leymus chinensis*) is an important C3 perennial cool-season forage grass widely distributing on the eastern Eurasian steppe (Bai et al., 2004). The tender tillers of sheepgrass are rich in cellulose, hemicellulose, and soluble proteins that can be utilized as a high-quality feedstock for ruminant animals such as cattle and sheep (Chen et al., 2015). Moreover, sheepgrass has a relative high forage yield that can reach 3.0–6.0 ton/hm^2^ dry matter biomass in the Northeastern Plain and east of the Inner Mongolian Plateau (Chen et al., 2002). Because of the economic importance of sheepgrass, the utilization and improvement of sheepgrass feedstocks have been broadly studied during the past decades (Chen et al., 2002, 2015; Wang et al., 2009). A good balanced diet for cows has been achieved by replacing part of corn silage and alfalfa hay with sheepgrass hay (Yan et al., 2011). However, the forage digestibility is highly variable in different sheepgrass germplasms (Xu and Zhou, 2006). It has been indicated that cell wall lignification extent of feedstock is one of the major factors influencing forage digestibility (Chen et al., 2002; Chen and Dixon, 2007). Therefore, the structural and compositional elucidation of sheepgrass internodes will provide a baseline understanding of relationships between cell wall lignification and digestibility.

Lignin together with cellulose and hemicellulose are the most abundant components of plant cell wall. Lignin covalently crosslinks with hemicellulose to form a compact structure embedding cellulose in secondary cell wall, which plays crucial roles in mechanical support, water transport, and plant defense during plant growth and development (Boerjan et al., 2003). Grass lignin polymers consist of three prevalent units, *p*-hydroxyphenyl (H), guaiacyl (G), and syringyl (S) (Hatfield et al., 1999b; Ralph et al., 2004). However, the proportions of lignin units are varied depending on plant species, tissue/organ types, and developmental stages (Boerjan et al., 2003). Previous studies have suggested that lignin impacts on forage digestibility by impairing the accessibility of rumen microbes to cellulose and hemicellulose (Wilson and Mertens, 1995; Chen et al., 2002). Therefore, conventional and molecular breeding have been employed to select elite forage cultivars or materials with both low lignin content and strong agronomic traits for many years (Cardinal et al., 2003; Sattler et al., 2010). Additionally, lignin modification in forage crops can dramatically improve the cattle’s intake and cell wall digestibility as well (Eudes et al., 2014). Furthermore, a strong negative correlation between lignin content and saccharification efficiency of cell wall has been observed in stems of tall fescue, alfalfa, and switchgrass (Chen et al., 2002; Chen and Dixon, 2007; Shen et al., 2009). The culms consisting of a series of successive internodes account for >50% of above-ground dry matter biomass of sheepgrass (Chen et al., 2002). Surprisingly, little information is available on structure features, cell wall composition, and lignification patterns of sheepgrass culms.

Monolignol biosynthetic pathway comprises a highly coordinated set of metabolic events that include at least 10 enzyme gene families (Boerjan et al., 2003; Zhao and Dixon, 2011). The successive internodes at different developmental stages are spatially separated along the culm in grass family plants and provide an excellent system for molecular analysis of cell wall lignification. Previous studies have shown that the expression of monolignol biosynthetic pathway genes is under spatial and temporal control during internode development (Riboulet et al., 2009; Shen et al., 2009; Zhang et al., 2014; McKinley et al., 2016). Generally, lignin-related genes have high expression levels in tissues undergoing active lignification. However, not all lignin-related genes exhibit a similarly temporal and spatial transcript profile during the process of internode lignification, suggesting a complex regulation mechanism in monolignol biosynthetic pathway (Shen et al., 2009; Zhao and Dixon, 2011). Compared with other forage and agriculturally important crops in the Poaceae family, the molecular genetic information is currently elusive for the developmental control of lignification in sheepgrass.

Sheepgrass has emerged as a source of drought, cold, saline, and alkaline resilient forage grass (Huang et al., 2004). Recent studies on sheepgrass have focused on how to utilize this type of lignocellulosic feedstock and its gene resource (Wang et al., 2009; Chen et al., 2015; Gao et al., 2016; Zhao et al., 2016). In our work, sheepgrass internodes at different developmental stages were collected for investigating the effects of lignification on cell wall digestibility. The results showed that the structural features, lignin-related gene expression profiles, and lignification patterns of internodes were significantly altered with internode maturation in sheepgrass. Further studies revealed a strong negative correlation between lignin and cell wall digestibility of sheepgrass. These data provide a baseline information for utilization and improvement of sheepgrass feedstock in future.

## Materials and Methods

### Plant Materials and Sample Collection

A highly productive sheepgrass cultivar, Nongjing No. 4, was employed for structural and compositional analysis of internodes. Sheepgrass plants were grown in the greenhouse with 16 h light (390 μE m^-2^ S^-1^). The development of sheepgrass in our green house was divided into three vegetative stages (V1, V2, and V3), six elongation stages (E1, E2, E3, E4, E5, and E6), and three reproductive stages (R1, R2, and R3) according to the criteria described by Moore et al. (1991). Internode 2 was collected from the corresponding tillers at different elongation stages (E2, E3, and E4), respectively. Leaf blades, leaf sheaths, and culms were separated from the tillers harvested at the R1 stage, and then the successive internodes (I1-6) were dissected from the R1 culms. The above samples were immediately frozen in liquid nitrogen and stored at -80°C for further analysis.

### Microscopy and Histochemical Assay

The middle portion of each internode of sheepgrass was cut into 30 μm sections with a vibratome 1000 (Ted Pella Inc., Redding, CA, USA) for internode structure and lignification analysis. Phloroglucinol-HCl staining and Mäule staining were carried out to determine lignin deposition in cell walls as described (Chen et al., 2002). The micrographs were taken under the Olympus SZX16 system with an Olympus DP72 color camera (Olympus, Tokyo, Japan).

### Determination of Lignin-Related Gene Expression Profiles

Total RNAs were extracted from sheepgrass internodes using Trizol reagent (Invitrogen, Chicago, IL, USA) according to the manufacturer’s instructions and were subjected to reverse transcription with One-step reverse transcription kit (Transgen, Beijing, China) after treatment with Turbo DNase I (Ambion, Austin, TX, USA). The sequences of 10 enzyme genes in monolignol biosynthetic pathway previously published in Brachypodium, rice, maize, and switchgrass were downloaded from Phytozome (Vogel et al., 2006; Riboulet et al., 2009; Ambavaram et al., 2011; Shen et al., 2013). The degenerate primers for each gene were designed by aligning the sequences from the above organisms. The fragments of *PAL, C4H, HCT, C3H, 4CL, CCR, CCoAOMT, COMT, F5H*, and *CAD* were amplified by reverse transcription polymerase chain reaction (RT-PCR) from sheepgrass and subjected to sequencing.

Quantitative real-time PCR (qRT-PCR) was employed to measure expression levels of lignin-related genes in sheepgrass internodes. The specific qRT-PCR primers were designed based on the above fragments isolated from sheepgrass, and expression profiles of lignin-related genes were determined (Supplementary Table S1). SYBR Green (Takara, Dalian, China) was used as the reporter dye. The cycle thresholds were determined using the LightCycler^®^ 480 Real-time PCR System (Roche Applied Science, Upper Bavaria, Germany), and the data were normalized using the level of sheepgrass *ACTIN* transcripts (Gao et al., 2016).

### Determination of Monosaccharide Composition and Cellulose Content

The collected samples were ground in liquid nitrogen and lyophilized. To obtain sufficient internode materials at the R1 stage for biochemical analysis, we pooled every two successive internodes (I1/2, I3/4, and I5/6) along the culm. The extractive-free cell wall residues (CWRs) were prepared from the above lyophilized materials as described by Chen and Dixon (2007) and used for cell wall composition analysis.

Matrix polymers were extracted from sheepgrass CWRs with 2 mol/l trifluoroacetic acid (TFA) at 121°C for 2 h. Monosaccharide composition of the above matrix polymers was analyzed as described (Tang et al., 2015). The monosaccharides of each of sample were identified and quantified by high performance liquid chromatography (HPLC) with precolumn-derivatization based on their corresponding standard compounds. Cellulose content of sheepgrass CWRs was analyzed as described (Tang et al., 2015). The pellets after extraction of matrix polymers were hydrolyzed in 72% sulfuric acid at 30°C for 30 min, and then the released glucose content was analyzed spectrophotometrically using the phenol-sulfuric acid assay method (Dubois et al., 1956). The numerical value of glucose content multiplied by 0.9 represents cellulose content.

### Determination of Lignin Content and Composition

The acetyl bromide (AcBr) method described by Hatfield et al. (1999a) was used to quantify lignin content. Lignin composition was determined by the thioacidolysis method (Lapierre et al., 1995). The samples were analyzed by a Hewlett-Packard 5890 series II gas chromatograph with a 5971 series mass selective detector (HP-1 column, 60 m × 0.25 mm × 0.25 μm film thickness). Mass spectra were recorded in electron impact mode (70 eV) with 60–650 m/z scanning range (Fu et al., 2011a). Lignin units were identified and quantified by characteristic mass spectrum ions of 239 m/z (H), 269 m/z (G), and 299 m/z (S).

### Determination of Saccharification Efficiency

Saccharification efficiency of sheepgrass CWRs was measured as described (Fu et al., 2011a). Sugar release was detected with the phenol-sulfuric acid assay method (Dubois et al., 1956). Saccharification efficiency was determined as the ratio of sugars released by enzymatic hydrolysis to the amount of sugars present in cell wall materials before pretreatment.

### Statistical Analysis

Triplicate samples were collected for each developmental stage. The mean values were used for statistical analysis. Data from each trait were subjected to one-way analysis of variance (ANOVA). The significance of treatments was tested at the *p* < 0.05 level. Standard errors are provided in all figures and tables as appropriate. Spearman correlation coefficients were determined between saccharification efficiency and cell wall component contents. All correlations with *p* < 0.05 were treated as correlated.

## Results

### Cell Wall Features of Sheepgrass Internodes

**FIGURE 1:**
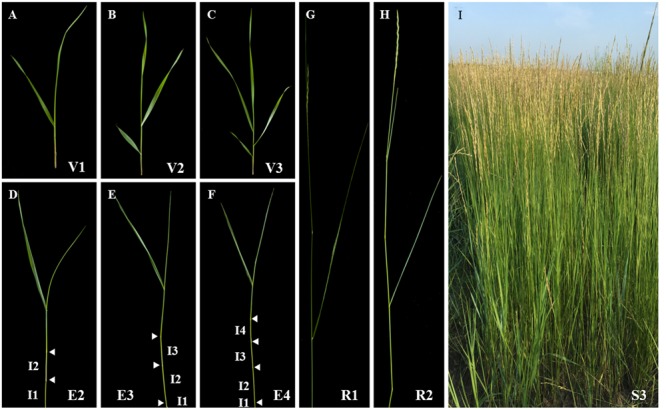
**Developmental stages of sheepgrass shoots.** Various vegetative (**A–C**: V1-3), elongation (**D–F**: E2-4), reproductive (**G,H**: R1-2), and seed ripening (I: S3) stages of sheepgrass tillers were identified as described by Moore et al. (1991) and photographed. Arrowheads indicate visible nodes. Internodes are labeled on the tillers at E2, E3, and E4 stages. I1: internode 1; I2: internode 2; I3: internode 3; I4: internode 4.

The sheepgrass plant is a collection of tillers at various developmental stages. Each tiller consists of a series of phytomers including leaf blade, leaf sheath, node, internode, and axillary bud. Therefore, accurate identification of the growth stage of tillers can facilitate making good decisions in establishment, grazing management, harvesting, and seed production of sheepgrass. Based on the nomenclature of tiller stages described by Moore et al. (1991), we divided the life cycle of sheepgrass tillers into vegetative, elongation, reproductive, and seed ripening stages, which are associated with a progressive lignification of cell walls (**Figure 1**). Among them, the lignification extent of tillers at late elongation or early reproductive stage determines the forage digestibility of sheepgrass. Thereby, we next studied the process of cell wall lignification of internodes with tiller development.

**FIGURE 2:**
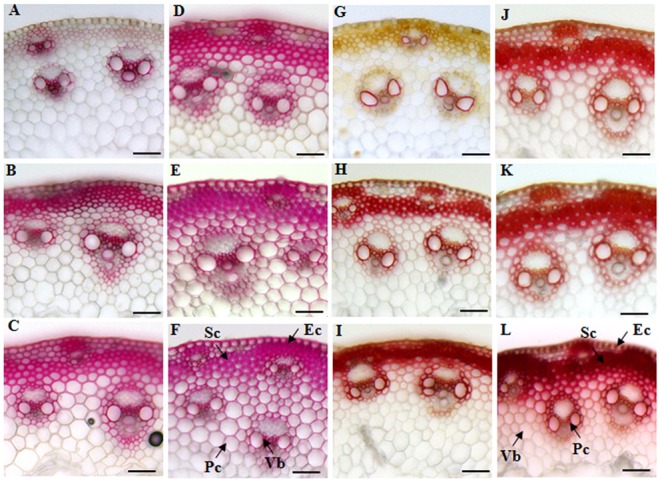
**Histochemical analysis of lignin in cross-sections of sheepgrass internodes.** Internodes 2 at E2, E3, E4 stages and the internodes (I2, I4, and I6) along the culm at the R1 stage were collected. The middle portions of each internode were cut into 30-μm transverse sections. **(A–F)** Phloroglucinol-HCl staining of E2-I2, E3-I2, E4-I2, R1-I6, R1-I4, and R1-I2. Red coloration roughly reflects the total lignin content; **(G–L)** Mäule staining of E2-I2, E3-I2, E4-I2, R1-I6, R1-I4, and R1-I2; G lignin units are colored in orange, and S lignin units are colored in red. Sc, sclerenchyma; Pc, parenchyma; Ec, epidermal cells; Vb, vascular bundle; Bar = 100 μm.

We first decided to examine the cell wall features of different tissues in elongating internodes. The cross-sections of internode 2 from different developmental stages were stained with phloroglucinol-HCl and Mäule reagents that can give an indication of lignin deposition. Both phloroglucinol-HCl and Mäule staining assay showed that lignification and wall thickness of epidermal, parenchyma, sclerenchyma, and xylem cells were progressively enhanced with internode maturation (**Figures 2A–C,G–I**). Compared with xylem, phloem constitutively maintained a weak lignification status during vascular bundles development, suggesting a strict regulation on lignification in tissues with distinct functions. Moreover, a marked increase of cell wall lignification and thickening in interfascicular fibers and vascular bundles was observed when the internode reached maturity (**Figures 2C,I**). Furthermore, we studied the cell wall characterization of successive internodes along the culm at the R1 stage. A similar trend of cell wall lignification and thickening was found from top internode (I6) to basal internode (I2) along the sheepgrass culm (**Figures 2D–F,J,K**). Additionally, an apparent red coloration which is diagnostic of S lignin units with Mäule reaction was specially exhibited in interfascicular fiber and sclerenchyma sheath surrounding the vascular bundles during internode development.

### Expression Profiles of Lignin-Related Genes in Sheepgrass Internodes

**FIGURE 3:**
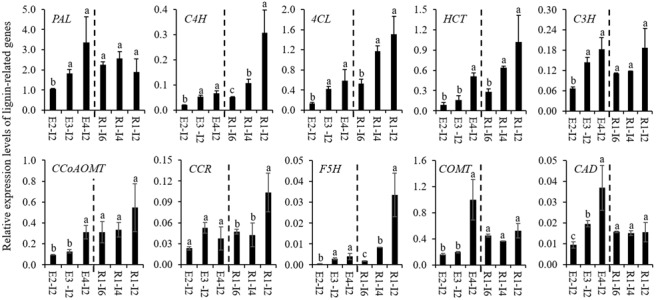
**Expression profiles of lignin-related genes in sheepgrass.** Internodes 2 at E2, E3, E4 stages and the internodes (I2, I4, and I6) along the culm at the R1 stage were collected from sheepgrass tillers. The expression levels of lignin-related genes from monolignol biosynthetic pathway in sheepgrass were revealed by quantitative real-time PCR (qRT-PCR). Sheepgrass *ACTIN* was used as the reference for normalization. Values are means ± SE (*n* = 3). Means with the different letter are significantly different (One-way ANOVA, Duncan’s test, *p* < 0.05).

To gain an insight into the molecular mechanism of cell wall lignification, 10 lignin-related genes were isolated from sheepgrass and their expression profiles were studied. Due to little molecular genetic information available for sheepgrass lignin biosynthesis, we isolated the fragments of lignin-related genes from sheepgrass mainly based on their homologs from switchgrass, of which most of these genes have been functionally verified to participate in lignin biosynthesis (Shen et al., 2013). The deduced amino acid sequences of sheepgrass PAL, C4H, 4CL, HCT, C3H, CCoAOMT, CCR, F5H, COMT, and CAD fragments shared 87.5, 83.2, 86.1, 88.3, 88.8, 85.0, 88.3, 79.8, 87.0, and 89.0% similarity with their corresponding homologs from switchgrass, respectively. Furthermore, the results of qRT-PCR analysis revealed that the expression levels of lignin-related genes except *CCR1* were significantly upregulated when the internode reached maturity (**Figure 3**). The internodes at the R1 stage representing the gradual process of cell wall lignification were separated by removing leaf, sheath, and node. The expression profiles of lignin-related genes in these internodes were further clustered into two groups. The expression profiles of group I genes (*C4H, 4CL, HCT, CCR*, and *F5H*) roughly followed the pattern of cell wall lignification of internodes, whereas group II genes (*PAL, C3H, CCoAOMT, COMT*, and *CAD*) were expressed at a stable level from internode 6 to internode 2 (**Figure 3**).

### Cell Wall Composition of Sheepgrass

The main components of sheepgrass cell walls included cellulose, hemicellulose, and lignin. Cellulose and lignin contents of leaf blade were much lower than those of leaf sheath and culm in sheepgrass (**Tables 1, 2**). The dynamics of accumulation of cell wall components were further analyzed during sheepgrass internode development. Our results showed that the content of cellulose and monosaccharides remained stable in internode maturation (**Table 1**). However, AcBr lignin content was increased by 11.1% in the mature internode (E4-I2) compared with the elongating internode (E2-I2) (**Table 2**). After the E4 stage, the elongation of internode 2 had ceased, and then lignin was incorporated into secondary cell walls at a steady rate in sheepgrass. Thus, lignin content of R1-I2 was slightly increased compared with that of E4-I2 (**Table 2**). Furthermore, lignin composition was evaluated by the thioacidolysis yield of lignin monomers that represents the relative content of lignin monomers bound by β-O-4 linkages. Our lignin composition analysis showed that G lignin units had a similar accumulation pattern with that of AcBr lignin content, whereas H lignin levels were stable during internode development. It is noteworthy that S lignin units exhibited a continuous increase in internode maturation, and as a consequence the ratio of S/G rose gradually in these internodes (**Table 2**). The accumulation patterns of lignin content and compositions observed in the internodes at different developmental stages were further confirmed in the successive internodes along the culm at the R1 stage (**Table 2**). Moreover, our lignin content and composition data were consistent with cell wall characterization analysis by microscopy and lignin staining as mentioned above (**Figure 2** and **Table 2**).

**Table 1:** Monosaccharide composition of non-cellulosic cell wall carbohydrates in sheepgrass.^∗^

Organs	Cellulose (mg/g CWR)	Xylose (mg/g CWR)	Arabinose (mg/g CWR)	Glucose (mg/g CWR)	Mannose (mg/g CWR)	Galactose (mg/g CWR)	Rhamnose (mg/g CWR)	Galacturonic acid (mg/g CWR)
E2-I2	448.5 ± 9.5^a^	82.6 ± 0.40^a^	12.3 ± 0.50^a^	3.9 ± 0.21^a^	2.7 ± 0.92^a^	0.8 ± 0.11^a^	0.38 ± 0.033^a^	2.0 ± 0.43^a^
E3-I2	457.8 ± 13.4^a^	72.5 ± 7.22^a^	10.6 ± 0.86^b^	3.1 ± 0.36^b^	2.2 ± 0.33^a^	0.7 ± 0.20^a^	0.33 ± 0.021^a^	1.4 ± 0.20^b^
E4-I2	452.6 ± 3.8^a^	77.0 ± 2.91^a^	11.1 ± 0.55^a^	3.9 ± 0.28^a^	2.7 ± 0.66^a^	0.8 ± 0.02^a^	0.36 ± 0.036^a^	1.5 ± 0.27^b^
R1-I2	456.8 ± 1.3^a^	84.2 ± 1.59^a^	7.1 ± 0.16^a^	3.9 ± 0.13^b^	2.7 ± 0.26^a^	1.0 ± 0.05^a^	0.41 ± 0.016^b^	0.8 ± 0.14^a^
R1-I4	472.1 ± 32.0^a^	84.8 ± 1.54^a^	8.2 ± 0.13^a^	4.2 ± 0.14^b^	2.7 ± 0.21^a^	1.1 ± 0.01^a^	0.40 ± 0.018^b^	1.0 ± 0.03^a^
R1-I6	499.1 ± 12.5^a^	83.3 ± 2.19^a^	7.8 ± 0.07^a^	5.2 ± 0.23^a^	0.7 ± 0.48^b^	1.1 ± 0.06^a^	1.52 ± 0.10^a^	0.8 ± 0.02^a^
Leaf blade	411.9 ± 28.3^b^	79.9 ± 1.83^b^	9.1 ± 0.31^b^	3.7 ± 0.21^a^	1.8 ± 0.04^b^	1.6 ± 0.07^a^	0.43 ± 0.018^a^	2.4 ± 0.21^a^
Leaf sheath	507.4 ± 4.3^a^	82.4 ± 0.45^ab^	9.8 ± 0.10^c^	4.0 ± 0.23^a^	1.6 ± 0.17^b^	1.4 ± 0.06^b^	0.39 ± 0.057^a^	2.4 ± 0.11^a^
Culm	487.0 ± 2.7^a^	84.8 ± 0.56^a^	6.9 ± 0.13^a^	3.6 ± 0.09^a^	2.6 ± 0.21^a^	0.8 ± 0.01^c^	0.42 ± 0.035^a^	2.2 ± 0.21^a^

*^∗^Internodes at E2, E3, E4, and R1 stages were collected, and every two successive internodes (I1/2, I3/4, and I5/6) along the culm at the R1 stage were pooled. Values are mean ± SE (*n* = 3). Means with the different letter are significantly different (One-way ANOVA, Duncan’s test, *p* < 0.05).*

**Table 2:** Lignin content and composition of sheepgrass.^∗^

		Thioacidolysis yield (μmol/g CWR)			
Organs	AcBr lignin (mg/g CWR)	H units	G units	S units	S/G
E2-I2	151.7 ± 1.78^b^	33.0 ± 1.29^a^	212.3 ± 5.37^b^	97.2 ± 5.11^b^	0.46
E3-I2	157.6 ± 3.56^ab^	33.6 ± 0.73^a^	240.6 ± 2.98^a^	129.3 ± 2.34^ab^	0.54
E4-I2	168.5 ± 3.62^a^	32.0 ± 0.64^a^	243.0 ± 8.40^a^	145.9 ± 2.19^a^	0.60
R1-I5/6	148.8 ± 2.28^c^	21.6 ± 7.27^a^	186.8 ± 26.08^b^	122.7 ± 20.38^b^	0.66
R1-I3/4	160.9 ± 0.06^b^	30.7 ± 3.77^a^	233.2 ± 1.13^ab^	163.6 ± 1.11^ab^	0.70
R1-I1/2	169.9 ± 0.57^a^	36.0 ± 1.52^a^	250.8 ± 11.36^a^	181.2 ± 9.00^a^	0.72
Leaf blade	118.9 ± 2.45^c^	7.0 ± 0.01^b^	85.3 ± 0.01^b^	34.5 ± 0.04^c^	0.40
Leaf sheath	151.6 ± 1.21^b^	24.6 ± 1.00^a^	214.5 ± 2.72^a^	111.2 ± 1.18^b^	0.52
Culm	162.7 ± 0.16^a^	24.8 ± 0.68^a^	214.1 ± 0.05^a^	149.3 ± 0.49^a^	0.70

*^∗^Internodes at E2, E3, E4, and R1 stages were collected, and every two successive internodes (I1/2, I3/4, and I5/6) along the culm at the R1 stage were pooled. Values are mean ± SE (*n* = 3). Means with the different letter are significantly different (One-way ANOVA, Duncan’s test, *p* < 0.05).*

### Relationship between Saccharification Efficiency and Cell Wall Components

**FIGURE 4:**
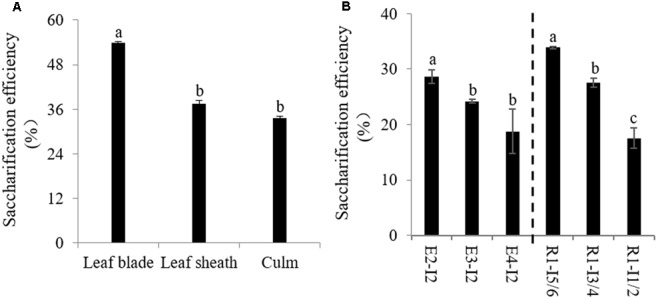
**Saccharification efficiency of sheepgrass.** Internodes at E2, E3, E4, and R1 stages were collected, and every two successive internodes (I1/2, I3/4, and I5/6) along the culm at the R1 stage were pooled. **(A)** Saccharification efficiency of different internodes at E2, E3, E4, and R1 stages; **(B)** Saccharification efficiency of leaf blade, leaf sheath, and culm at the R1 stage; values are means ± SE (*n* = 3). Means with the different letter are significantly different (One-way ANOVA, Duncan’s test, *p* < 0.05).

We next decided to access the impact of lignification on cell wall digestibility since the amount of solubilized sugar released from cell wall is the critical factor affecting the utilization of sheepgrass feedstock. We first determined saccharification efficiency of cell walls of leaf blade, leaf sheath, and culm biomass from sheepgrass tillers at the R1 stage. Our data revealed that cell wall polysaccharides of leaf blade were apt to be easily degraded by cellulase compared with leaf sheath and culm (**Figure 4A**). There were up to 18 and 20% increases in saccharification efficiency from leaf blade biomass relative to leaf sheath and culm biomass, respectively (**Figure 4A**). Furthermore, we examined the effect of internode lignification on cell wall digestibility because culm can occupy more than 50% dry matter biomass of sheepgrass feedstock at each harvest. The results clearly showed that the saccharification efficiency of cell wall polysaccharides was gradually declined with internode maturation (**Figure 4B**).

**FIGURE 5:**
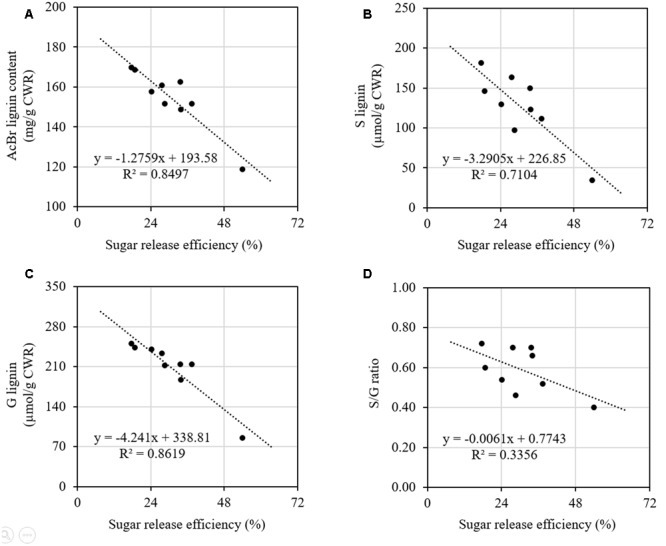
**Relationships between saccharification efficiency and lignin content and composition.** Data from **Figure 4** and **Table 2** were used for correlation analysis. **(A)** Correlation of saccharification efficiency and AcBr lignin content; **(B)** Correlation of saccharification efficiency and G lignin unit levels; **(C)** Correlation of saccharification efficiency and S lignin unit levels; **(D)** Correlation of saccharification efficiency and S/G ratios. Spearman correlation coefficients were determined between saccharification efficiency and lignin content and composition.

We further did correlation analysis to gain more insights on the effects of cell wall components on digestibility with different types of sheepgrass biomass including leaf blade, leaf sheath, culm, and internodes at different developmental stages. Our results revealed a strong negative correlation between AcBr lignin content and saccharification efficiency (**Figure 5A**). In contrast, neither cellulose nor xylose was correlated with saccharification efficiency of sheepgrass (**Supplementary Figure S1**). In addition, both G and S lignin unit levels were inversely correlated with saccharification efficiency (**Figures 5B,C**). However, saccharification efficiency was not correlated with H unit levels or S/G ratio in sheepgrass (**Figure 5D** and **Table 2**).

## Discussion

As one of important forage sources, sheepgrass has relative lower lignin accumulation and strong tolerance to drought, cold, saline, and alkaline conditions (Huang et al., 2004). However, little information is available on lignin biosynthesis of sheepgrass. The internode structures and associated lignin-related gene expression profiles and lignin accumulation patterns were comprehensively investigated for the first time in sheepgrass, which indicate that cell wall lignification rose progressively with internode maturation under a spatial and temporal regulation pattern. Previous studies on the distribution of lignin units also suggest that G units deposit in xylem cells dominantly, whereas both G and S units are present in interfascicular fiber cells in *Arabidopsis* and *Medicago* (Chapple et al., 1992; Nakashima et al., 2008). In contrast, our histochemical assay of sheepgrass internode suggests that xylem cells also contained abundant S lignin units. The molecular mechanism that leads to a wide distribution of S lignin units in different tissues of sheepgrass is currently unknown, but it is worth additional investigations in future.

To date, the entire lignin biosynthetic pathway has yet to be drawn in monocot species. Although the dynamics of lignin-related gene expression during internode development have been studied in switchgrass, maize, and sorghum, only a few genes have been functionally characterized *in vitro* or *in vivo* (Riboulet et al., 2009; Shen et al., 2009; Sattler et al., 2010; McKinley et al., 2016). In the present work, we systematically analyzed the expression profiles of lignin-related genes in sheepgrass and found that most lignin-related genes were highly expressed in well-lignified internodes. Thus, the expression profile analysis of lignin-related genes in sheepgrass internodes may provide a simple and rapid preliminary method to seek the candidate targets for genetic improvement of sheepgrass with low lignin and high forage digestibility. However, not all transcript profile of lignin-related genes exactly followed the pattern of lignin accumulation during sheepgrass internode development. Based on our current understanding of monolignol biosynthesis, the apparent inconsistence between expression profile of group II genes and internode lignification pattern could be explained by the possible roles of group II genes in the biosynthesis of other related metabolites such as flavonoids, caffeic acid, and ferulic acid in sheepgrass. It has been suggested that lignin reduction can result in a substantial decrease in quercetin, caffeic acid, and sinapoyl malate in *Arabidopsis omt1* mutant (Goujon et al., 2003). Another alternative explanation could be involved in a possible modulation on the stability and activity of enzymes encoded by group II genes. Indeed, the previous studies have indicated that the ubiquitination and subsequent degradation of PAL can be affected by the Kelch domain-containing F-box proteins in *Arabidopsis* (Zhang et al., 2013). Moreover, COMT enzyme activity in poplar monolignol biosynthesis can be switched on/off via a phosphorylation-mediated regulation model (Wang et al., 2015).

Forage digestibility is mainly determined by the total amounts of cell wall polysaccharides and their degradation efficiency by livestock. Cellulose, hemicellulose, and lignin are critical factors affecting forage digestibility and livestock industry. In agreement with the previous studies (Chen et al., 2002; Jung and Casler, 2006; Lingle and Thomson, 2012; Rancour et al., 2012; Zhang et al., 2013), a continuous increase in lignin accumulation was observed in internode 2 from the E2 to E4 stage and the successive ones along the culm at the R1 stage as well. Moreover, an impressive shifting of lignin composition was observed in internodes at late stages in sheepgrass, implying that S lignin units may play an important role in cell wall strengthen and mechanical support of culms. Besides the developmental stages of materials, the proportions of lignin are various depending on plant species and tissue/organ types. Compared with other forage grass or silage such as switchgrass, maize, and sorghum, sheepgrass tillers have a relative low lignin content and S/G ratio (Jung and Casler, 2006; Li et al., 2014; Wu et al., 2016; **Table 1**). In addition, cellulose and lignin contents of leaf blade were much lower than those of leaf sheath and culm in sheepgrass. It can be explained by the fact that leaf blade mainly consists of mesophyll cells while leaf sheath and culm comprises more sclerenchyma cells. Thus, supportive tissues exhibited a greater extent of lignification than mesophyll parenchymous tissues in sheepgrass.

Lignin present in cell walls, however, is one of crucial factors impairing lignocellulosic biomass utilization. Previous studies have demonstrated that saccharification efficiency can roughly reflect the extent of forage digestibility and lignocellulosic biomass bioconversion (Chen et al., 2002; Chen and Dixon, 2007; Shen et al., 2009; Fu et al., 2011a,b; Xu et al., 2011). Our results indicate that both lignin content and the major lignin units (G and S) levels can significantly affect the degradation of cell wall polysaccharides in sheepgrass, suggesting that both polysaccharides yield and lignification extent of cell walls need to be considered in sheepgrass feedstock utilization. However, we cannot clarify the detailed contribution of lignin content and lignin composition to cell wall digestibility. Previously, Chen and Dixon (2007) have identified lignin content but not lignin composition as the probable major factor in recalcitrance of cell walls to saccharification by using various transgenic alfalfa lines. In contrast, a strong negative correlation between lignin composition and forage digestibility or saccharification efficiency has been observed in tall fescue and switchgrass, respectively (Chen et al., 2002; Shen et al., 2009). Additionally, it should be noted that other factors including wall-bound phenolics, cellulose and hemicellulose structures can contribute together to cell wall digestibility of forage and biofuel feedstocks. Thus, generation of transgenic sheepgrass plants with various lignin composition and minimum side effects from other cell wall components could be a desirable strategy for investigating the cause effect of lignin properties on cell wall degradation in future.

## Author Contributions

JW, ZS, DS, and CF designed the research. JW, LM, PZ, ZB, HZ, YC, and YB performed the experiments. CF, JW, LM, ZS, and DS analyzed the data. CF and LM wrote the article.

## Conflict of Interest Statement

The authors declare that the research was conducted in the absence of any commercial or financial relationships that could be construed as a potential conflict of interest.

## Supplementary Material

FIGURE S1 **Relationships between saccharification efficiency and cellulose and xylose contents.** Data from **Figure 4** and **Table 1** were used for correlation analysis. **(A)** Correlation of saccharification efficiency and cellulose content; **(B)** correlation of saccharification efficiency and xylose content. Spearman correlation coefficients were determined between saccharification efficiency and lignin content and composition.
