# Supplementary figures and images for: Lignification of Sheepgrass Internodes at Different Developmental Stages and Associated Alteration of Cell Wall Saccharification Efficiency
Source: Front Plant Sci. 2017 Mar 27;8:414. doi: 10.3389/fpls.2017.00414 (PMC5366342; doi:10.3389/fpls.2017.00414)

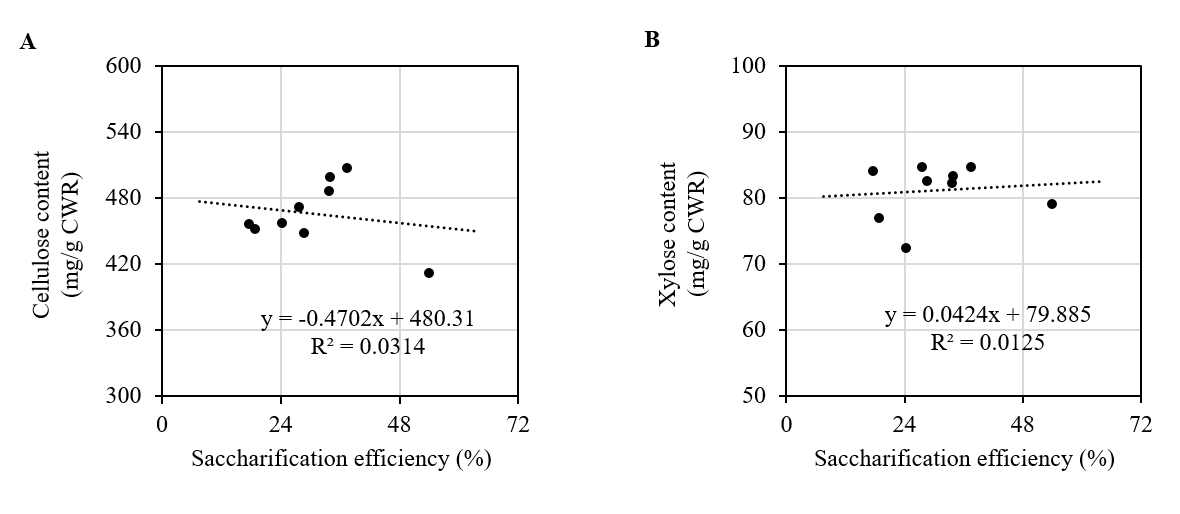

Supplement: FIGURE S1 — Relationships between saccharification efficiency and cellulose and xylose contents. Data from Figure 4 and Table 1 were used for correlation analysis. (A) Correlation of saccharification efficiency and cellulose content; (B) correlation of saccharification efficiency and xylose content. Spearman correlation coefficients were determined between saccharification efficiency and lignin content and composition. [file Image_1.TIF]
