# Supplementary material for: Lignification of Sheepgrass Internodes at Different Developmental Stages and Associated Alteration of Cell Wall Saccharification Efficiency
Source: Front Plant Sci. 2017 Mar 27;8:414. doi: 10.3389/fpls.2017.00414 (PMC5366342; doi:10.3389/fpls.2017.00414)
Supplement: Supplementary file 2 [file Table_1.DOC]

**Supplemental Table S1** **Primer sequences used for lignin-related gene expression analysis by qRT-PCR.**

| Gene | Primer | Gene | Primer |
| --- | --- | --- | --- |
|  |  |  |  |
| *PAL* | F: TCACCAAGCTCATCAACACC | *C4H* | F: ACGTTGTCGTGGTTGATCTC |
|  | R: CTTTCCGTCAACAGTAGTGG |  | R: AAGCGGATGAAGGTCTTCGA |
| *HCT* | F: ACTACACCGACGACATATCC | *4CL* | F: TCCACCATCCCACTTCTTAC |
|  | R: ACCACGAGTTGATGAAGTGC |  | R: CCCTGACTTCTTTAAGGCTG |
| *CCoAOMT* | F: ATCCTGGCCATGGACATCAA | *C3H* | F: GTAGTGAGGAATCACCTTGC |
|  | R: TCCACGAAGACGAAGTCGAA |  | R: AGCAACAGAGAGAGATGCAC |
| *CCR* | F: TGGTGTTCACGTCGTCCATC | *F5H* | F: CCAAAACCTACCTCACCTAC |
|  | R: TGCCGTAGCAGTACCAGTTC |  | R: GGCTGAAGAGTTTCATCACG |
| *CAD* | F: GCAACAAGAAGATCTGGTCC | *COMT* | F: GAGCTGGTACTATCTGAAGG |
|  | R: TCACCACGAACTTCTGATCG |  | R: GGAGCTTCTTGGTGATGATG |
|  |  |  |  |
